# Immune function in a free-living bird varies over the annual cycle, but seasonal patterns differ between years

**DOI:** 10.1007/s00442-012-2339-3

**Published:** 2012-05-06

**Authors:** Arne Hegemann, Kevin D. Matson, Christiaan Both, B. Irene Tieleman

**Affiliations:** Animal Ecology Group, Centre for Ecological and Evolutionary Studies, University of Groningen, P.O. Box 11103, 9700 CC Groningen, The Netherlands

**Keywords:** Ecoimmunology, Immunity, Life cycle, Seasonality, Trade-off

## Abstract

A central hypothesis of eco-immunology proposes trade-offs between immune defences and competing physiological and behavioural processes, leading to immunological variation within and among annual-cycle stages, as has been revealed for some species. However, few studies have simultaneously investigated patterns of multiple immune indices over the entire annual cycle in free-living birds, and none has investigated the consistency of seasonal patterns across multiple years. We quantified lysis, agglutination, haptoglobin, leukocyte profiles, and body mass in free-living skylarks (*Alauda arvensis*) through two complete annual cycles and within and between four breeding seasons. The skylarks’ annual cycle is characterised by annually repeated changes in energy and time budgets, social structure and diet. If trade-offs relating to these cyclic changes shape evolution, predictable intra-annual immune patterns may result. Alternatively, intra-annual immune patterns may vary among years if fluctuating environmental changes affect the cost–benefit balances of immune function. We found significant variation in immune indices and body mass across the annual cycle, and these patterns differed between years. Immune parameters differed between four breeding seasons, and in all years, lysis and agglutination increased as the season progressed independent of average levels. Population-level patterns (intra-annual, inter-annual, within breeding season) were consistent with within-individual patterns based on repeated measurements. We found little evidence for sex differences, and only haptoglobin was correlated (negatively) with body mass. We conclude that immune modulation is not simply a pre-programmed phenomenon that reflects predictable ecological changes. Instead, fluctuating environmental conditions that vary among years likely contribute to the immunological variation that we observed.

## Introduction

The immune system is a major physiological component of self-maintenance and promotes survival by reducing the probability of disease-related mortality (Roitt et al. 1998). Because the immune system also incurs costs in terms of its production, maintenance and activation (Schmid-Hempel 2003; Klasing 2004), organisms likely adjust the amount of resources allocated to the system relative to other activities in order to maximize fitness. One of the central hypotheses in ecological immunology proposes that immune defences are traded off against competing physiological and behavioural processes (Sheldon and Verhulst 1996; Lochmiller and Deerenberg 2000; Norris and Evans 2000). If the outcome of this trade-off differs among annual-cycle stages depending on resource availability and/or fitness benefits, then seasonal modulations in immune function might result. Additionally, the selective pressures exerted by pathogens and parasites, which are also expected to shape these trade-offs (Horrocks et al. 2011), may differ spatially (Piersma 1997; Mendes et al. 2006) and are known to vary temporally (Dowell 2001; Cosgrove et al. 2008).

Seasonal variation in immune function has been described for a number of vertebrate taxa (reviewed by Nelson and Demas 1996; Nelson et al. 2002; Martin et al. 2008). This variation can manifest itself as an overall reduction in investment in the immune system or as a reallocation within the immune system (Lee 2006; Hasselquist 2007; Buehler et al. 2008a, b; Martin et al. 2008). Studies report reductions in immune indices during reproduction (e.g. Ilmonen et al. 2000; Bonneaud et al. 2003; Ardia 2005), migration (e.g. Owen and Moore 2006, 2008), moult (Martin 2005; Moreno-Rueda 2010) and winter (Svensson et al. 1998). Thus far, in free-living birds, most studies of immune function are restricted to part of an annual cycle. Only two studies have examined immune indices over more than two annual-cycle stages, and both found seasonal modulation of the immune system. In great tits (*Parus major*), the heterophil/lymphocyte ratio varies seasonally and peaks after breeding (Pap et al. 2010a). Among four annual-cycle stages, house sparrows (*Passer domesticus*) showed significant variation in six of eight measured immunological variables with patterns varying depending on the variable (Pap et al. 2010b).

Additional data on seasonal variation of different immune parameters over the complete annual cycle and data over multiple years are needed to determine if trade-offs lead to consistent intra-annual immune patterns. Two main hypotheses can be distinguished: (1) If seasonal patterns are due to trade-offs with other fitness-enhancing activities that are consistently cyclical, then seasonal immune modulation is predicted to be consistent across years. For example, a shift in energy allocated to reproduction, moult or migration can result in smaller energy investments into the immune system and can lead to seasonally-characteristic changes that are independent of year; (2) Alternatively or additionally, allocation to immune function could be a consequence of encountered environmental conditions that change not only throughout the year but may also vary between years (e.g. resource availability and pathogen pressure). Overall, relationships between annual-cycle stage and immune function may be inconsistent among years if inter-annual variation in environmental conditions is strong or if seasonal trade-offs are weak. Such high flexibility would indicate broad reaction norms of immune function (see also Pedersen and Babayan 2011).

Beyond helping to disentangle the two main hypotheses outlined above, studying multiple complete annual cycles has an array of broader biological and methodological implications. For example, understanding within- and between-annual cycle variation is crucial when interpreting studies that investigate only a limited part of the annual cycle or a single year. Furthermore, such studies can help identify and explain immunological differences across annual-cycle stages and/or years, between the sexes and in relation to body condition. Males and females are hypothesised to differently allocate resources to their immune system since the sexes can differ in terms of energy expenditure and parasite exposure (Hasselquist 2007; Martin et al. 2008). Additionally it has been suggested that energy stores are a proximate mechanism for seasonal modulations in immune responses (Owen-Ashley and Wingfield 2006; Owen-Ashley and Wingfield 2007), but energy stores can change among annual-cycle stages and between years, depending on environmental conditions. Lastly, seasonal and annual patterns could arise because of changes in population composition or due to individual flexibility. Repeatedly measuring individuals within and among seasons and years can shed light on this possibility and will help validate single time-point measurements.

**Fig. 1:**
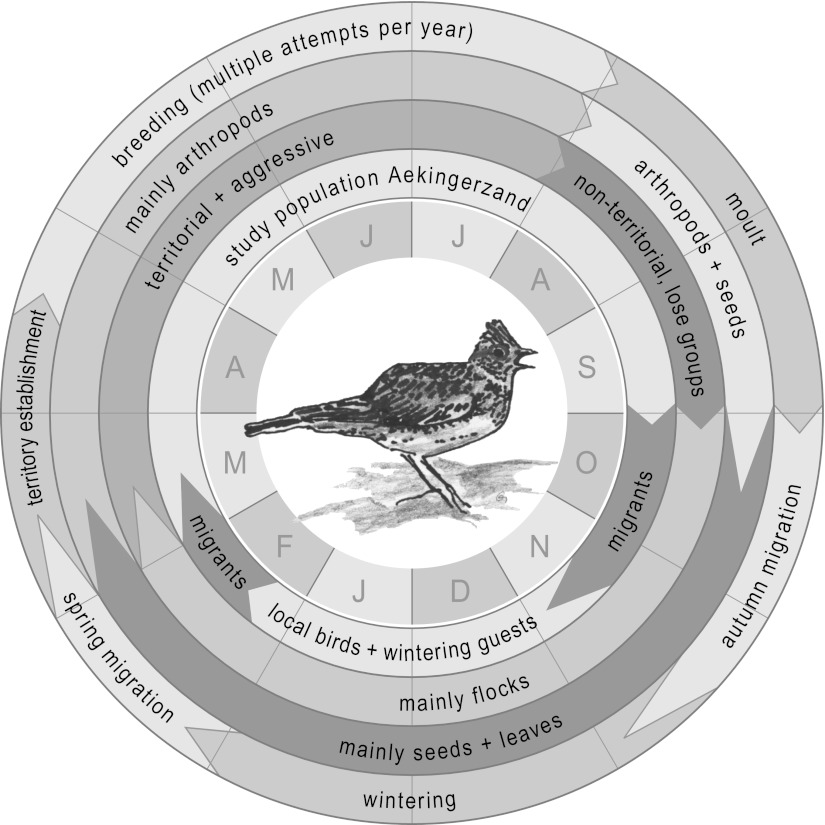
The annual cycle of a skylark (*Alauda arvensis*) in the northern Netherlands. Length of *arrows* in the transition between two stages indicates the amount of variability between years and/or individuals. Data on diet are based on Green (1978), Donald et al. (2001) and Geiger et al. (in preparation). All other data are based on our own unpublished data and Hegemann et al. (2010)

We tested these hypotheses on free-living skylarks (*Alauda arvensis*) by measuring components of the innate and acquired arms of the immune system across the entire annual cycle. Skylarks experience six distinct annual-cycle stages (territory settlement, breeding, moult, autumn migration, winter and spring migration), which vary in social structure, diet, activities, habitat choice (Fig. 1) and basal metabolic rate (Hegemann et al. 2012a). These stages could therefore represent differences in resources and in pressures from pathogens and parasites, affecting the trade-off for optimal investment in immune function and other fitness-enhancing traits.

We measured three general categories of immune defence: (1) the abilities of plasma to agglutinate and lyse foreign cells (Matson et al. 2005), processes which involve natural antibodies and complement and that are thought to be unaffected by previous exposure (Ochsenbein and Zinkernagel 2000); (2) acute phase protein (haptoglobin) concentrations, which usually increase in response to inflammation or infection (Thomas 2000; Matson et al. 2012); and (3) the relative abundances of five leukocyte types, which reflect both innate and acquired components and change in response to immunological stimulation (Feldman et al. 2000). The latter includes the ratio of heterophils and lymphocytes (H/L ratio) which is related to different types of stressors, including immunological ones (reviewed by Davis et al. 2008). We took biological and methodological factors into consideration when choosing to focus mainly on measures of innate immunity: This subsystem is an important first line of defence (Janeway et al. 2005), and this importance might translate into consistency over longer time scales, a point that coordinates with our main hypotheses. Additionally, while measures of innate immunity can vary over shorter scales (e.g. reflecting current “health status” or “physiological condition,” (Van de Crommenacker et al. 2010), the absence of immunological memory in vertebrate innate subsystems allows for interpretation of repeated samples without confounding the magnitude of an index and the exposure to a particular disease (Janeway and Medzhitov 2002; Kurtz 2004).

We explored three potential sources of variation in immune indices: (1) among annual-cycle stages, (2) between years, (3) within one annual-cycle stage (breeding). For all three categories, we also explored if, at the population level, body mass varies in a manner similar to the immune indices, and if, at the individual level, body mass correlates with these indices. Because sexes differ in behaviour and physiology, we included sex in our analyses. Lastly, we used intra-individual repeated measures to investigate whether seasonal and annual patterns within individuals were consistent with population mean seasonal and annual patterns. Because skylarks undergo characteristic seasonal changes in their ecology that are repeated on a regular basis, we expected corresponding intra-annual patterns of immune function that are repeatable among years, as a result of trade-offs between immune function and other physiological and behavioural demands. Alternatively, if fluctuating environmental conditions exert a substantial influence, then intra-annual patterns in immune function may differ among years. Fluctuating environmental conditions may affect immune patterns either qualitatively (i.e. the shape of seasonal patterns) or quantitatively (i.e. the magnitude of differences between seasons).

## Materials and methods

### Study system

Skylarks are widespread, temperate zone passerines that breed on the ground in open habitats ranging from natural steppes to modern agricultural farmland across Eurasia (Donald 2004). Birds from northern populations migrate, whereas southern populations are resident year-round, and western European birds are partial migrants (Glutz von Blotzheim and Bauer 1985; Donald 2004; Hegemann et al. 2010).

We studied skylarks throughout the annual cycle in the northern Netherlands (52°55′N, 6°18′E) in 2006–2009, focusing on a population in the Aekingerzand (Fig. 1). Skylarks in our study population are partial migrants. Some birds migrate, while others winter locally on agricultural fields that surround their breeding site, where they are accompanied by birds from northern and eastern Europe (Hegemann et al. 2010). During migration, birds from northern and eastern populations pass the Netherlands (LWVT/SOVON 2002). During all parts of the annual-cycle, skylarks forage and sleep exclusively on the ground.

We collected data during the breeding season in four consecutive years (2006–2009), and during two successive years (2007–2008) for moult, autumn migration, wintering and spring migration. We also collected samples during the territory establishment period in 1 year (2007). During territory establishment, breeding and moult, we sampled birds at the Aekingerzand. During territory establishment (27 February–22 April 2007), we lured birds (*n* = 14) into mist nets by using recordings of territorial songs and calls. During the four breeding seasons (21 April–3 August 2006–2009), we caught adults (*n* = 189) feeding nestlings with mist-nests or traps on the nest. During moult (14 August–27 September 2007; 2 August–22 September 2008), birds (*n* = 33) were caught at night by flushing them into nets. During winter (10 December 2007–22 January 2008; 9 December 2008–15 January 2009), we caught birds (*n* = 25) during the night by flushing them into nets on agricultural fields surrounding the Aekingerzand. During winter, we were unable to exclusively target birds from our study population, and we possibly caught a mix of local breeders and birds from more-northern breeding sites. During spring (14–24 March 2008; 25 February–1 March 2009; *n* = 27) and autumn (8 October–4 November 2007 and 11 October–2 November 2008, *n* = 97), actively migrating birds were caught during the day by luring them with conspecific songs to large clap-nets at a location 15 km southeast of the Aekingerzand. When tape-lured, migrants interrupt their migratory flight. Skylarks not actively migrating rarely respond to a tape lure during these seasons (Hegemann et al., unpublished observations); hence, we are confident about the migratory status.

Migrating and wintering birds were sampled only once, but a subset of skylarks was sampled repeatedly during other annual-cycle stages. We used these repeated measures to investigate whether seasonal and annual patterns at the population level were consistent with patterns at the individual level. During breeding, 44 birds were sampled twice; 19 birds, three times; 5 birds, four times; and 2 birds, six times. Repeated sampling occurred either within a single breeding season (*n* = 19) or in two (*n* = 38), three (*n* = 12) or four (*n* = 1) different breeding seasons. Some individuals were also caught in two different stages: 11 birds during territory establishment and breeding and 18 birds during breeding and moult.

### Sample and data collection

Blood samples (~100–150 μl) were collected into heparinised capillary tubes by puncturing the brachial vein with a sterile needle. Samples were collected immediately after capture (median 5 min; range 2–35 min) and before any expected impacts of handling stress (Buehler et al. 2008a). Blood smears for leukocyte enumeration were made from fresh blood. Blood was stored on ice until centrifuged in the laboratory (10 min, 7,000 rpm). Plasma and packed cell fractions were stored frozen for future analyses. Structural measurements (body mass, tarsus length, wing length) were taken after blood collection, and all birds were ringed with a metal ring from the Dutch Ringing Centre; birds from the study population Aekingerzand were additionally ringed with a unique combination of color rings. Birds were sexed biometrically and some doubtful cases were sexed molecularly (Hegemann et al. 2012b). All individuals included in this dataset were fully grown. Because skylarks undergo a complete post-nuptial moult in August–September, age classes could not be distinguished.

### Immune assays

We analysed the preserved plasma samples using two immunological assays. A hemolysis-hemagglutination assay was used to quantify titres of complement-like lytic enzymes and non-specific natural antibodies (Matson et al. 2005). Scans of individual samples were randomized among all plates and scored blindly to season, year and individual (by A.H.). A plasma standard was run in duplicate in all plates. The average within-plate variation (standard deviation) was 0.18 lysis titres and 0.32 agglutination titres. The average among-plates variation (calculated per batch) was 0.52 lysis titres and 0.80 agglutination titres. These values are only slightly higher than the variation originally described by Matson et al. (2005). A commercially available colorimetric assay kit (TP801; Tri-Delta Diagnostics, NJ, USA) was used to quantify haptoglobin concentrations (Matson et al. 2006). We followed the instructions provided by the kit manufacturer with a few minor modifications. Specifically, we extended the standard curve to a more diluted range. We also normalized all final haptoglobin values according to a plate-specific pool to control for variation within and among plates and batches (Matson et al. 2012). Both assays were carried out in four batches (July 2007, and February, March and September 2009). Samples from 2006 were analysed in batch 1, samples from the first annual cycle in batch 1 and 2, from the second annual cycle in batch 2 and 3 and samples from 2009 in batch 4.

Blood smears were prepared for microscopic observation (Campbell 1995) and smears were randomized and examined by one person (C. Gottland), who was blind to season. Leukocyte proportions were determined for the first 100 white blood cells (WBC) counted; in rare cases where cells were highly dispersed and WBCs were difficult to find, proportions were based on fewer than 100 cells. Cells where classified as lymphocytes, heterophils, basophils, monocytes or eosinophils (Latimer and Bienzle 2000).

### Data analysis and statistics

We present analyses of several data subsets: (1) two annual cycles (2007 and 2008) with five stages each (breeding, moult, autumn migration, wintering, spring migration), (2) one annual cycle (2007) that included a sixth annual-cycle stage (territory establishment), and (3) four breeding seasons (2006–2009). We defined an annual cycle (or bird year) as starting with territory establishment and ending with spring migration. We refer to these as 2006, 2007, 2008 and 2009 even though they differ slightly from calendar years.

We used linear mixed models and generalised linear models in the program R, v.2.9.2 (R Development Core Team 2009). Sex, annual-cycle stage and year and all interactions were included when applicable. To test if immune parameters are related to body mass at the individual level, we included a mass index (in g) in our models that was independent of season-, sex- and year-specific variation. We derived this mass index by calculating each individual’s deviation from the corresponding season-, sex- and year-specific population mean. Individual identity was included in all analyses as a random effect to avoid pseudo-replication. When comparing the four breeding seasons, we included Julian day and its square to test effects related to day length. Final models were achieved via backwards elimination (log likelihood ratio test, *P* < 0.05) using the “drop1”-function of *R*.

Data on white blood cells are only available for 2008 with the exception of spring migration where we have data from 2008 to 2009. *t *tests revealed that none of the WBC types differed between the two spring migrations (all *t* < 1.61, all *P* > 0.12), and thus data for this stage were pooled. We have no repeated measures for the WBC data. WBC data were analysed with generalized linear models with a quasi-binomial approach and *F* tests. White blood cell types were analysed separately using binomial approaches that incorporated the counts of one cell type and the total remaining WBC number (i.e. basophils against the sum of heterophils, lymphocytes, monocytes and eosinophils). Additionally, heterophils and lymphocytes were tested binomially as H/L ratio. Assumptions of all models were checked on the residuals of the final model.

For the plasma parameters and body mass for which we had repeated measures within individuals, we investigated whether patterns found within individuals were consistent with the average population level or were instead the result of other phenomena such as selective catching, individual differences in timing or changes in population composition. To accomplish this, we used the method described by van de Pol and Wright (2009) to test if population-level patterns differed from within-individual patterns. These analyses were restricted to within-breeding season patterns and the three annual-cycle stages (territory establishment, breeding and moult) with repeated measurements.

Significant interactions could result if large differences in annual means are paired with proportional changes among annual-cycle stages. If a year × annual-cycle stage interaction remained significant, we log-transformed response variables to investigate this possibility. In all cases, the interaction remained significant. To determine which annual-cycle stages (within year) and which breeding seasons (among years) differed from each other, we used Tukey posthoc tests (“multcomp” package; Hothorn et al. 2008).

## Results

### Variation among stages in a single annual cycle

**Fig. 2:**
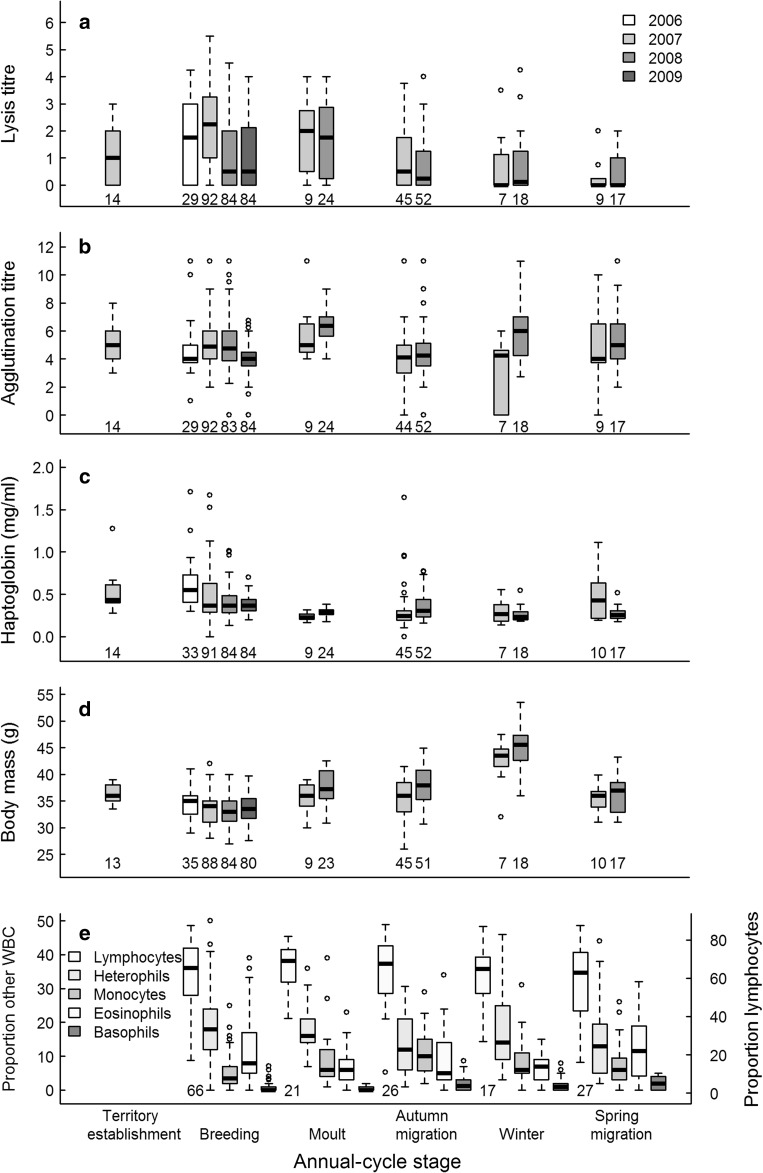
Seasonal patterns of **a** lysis, **b** agglutination, **c** haptoglobin concentrations, **d** body mass, and **e** the proportion of the different white blood cells (WBC, measured only in 2008) of free-living skylarks in six annual-cycle stages. Note that lymphocytes are scaled on the right *y* axis, while the other, less numerous, WBC types are scaled on the left *y* axis.* Numbers* under box-plots represent sample size. The *horizontal lines* in the box gives the median, the box covers the 25–75 % range, and *vertical lines* the 5–95 % range. *Dots* show outlying data points, which were included in the analyses

**Fig. 3:**
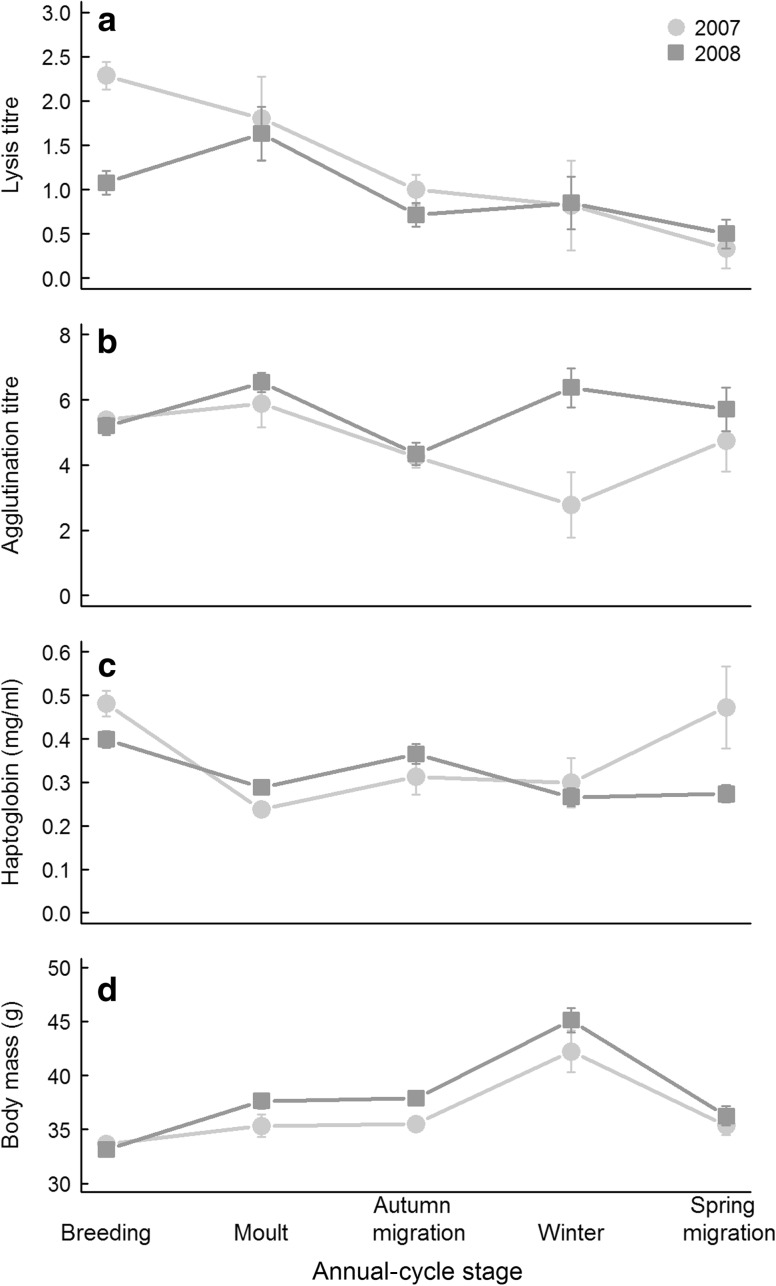
Patterns of average immune function throughout two annual cycles to illustrate the variation between immune patterns in two annual cycles. **a** Lysis titre, **b** agglutination titre, **c** haptoglobin concentrations and **d** body mass of skylarks. Shown are means with SE. (Note the difference from Fig. 2 where medians are shown)

Lysis, agglutination, haptoglobin and body mass varied among the six annual-cycle stages (2007) and three of the six cellular parameters (leukocyte profiles) varied among the five stages measured (2008) (Fig. 2a–e). Lysis peaked during breeding, agglutination declined from breeding to winter, and haptoglobin concentrations peaked during spring migration (Fig. 2a–c). Monocytes and basophils were highest during autumn migration, and eosinophils peaked during spring migration (Fig. 2e). Birds were heaviest during winter and lightest during breeding (Figs. 2d, 3d). Variation between annual-cycle stages was statistically significant for six immune parameters and body mass (Table 1). The proportions of heterophils and lymphocytes and the H/L ratio showed no significant seasonal variation (Table 1). Males had significantly higher lysis titres but a lower H/L ratio and lower proportions of heterophils than females throughout the annual cycle (Table 1). Males (37.14 ± 0.33 g, *n* = 94) were heavier than females (32.45 ± 0.25 g, *n* = 83).

**Table 1:** Statistics and coefficients of the linear mixed models (innate immune parameters and body mass) and generalized linear models (leukocyte profiles) of measures of immune function in skylarks (*Alauda arvensis*) throughout one annual cycle (2007 for innate immune parameters and 2008 for leukocyte profiles)

**Single annual cycle (2007 for innate immune parameters and 2008 for leukocyte profiles)**											
	Sex				Annual-cycle stage			BMd			
	*df*	*F*	β	*p*	*df*	*F*	*p*	*df*	*F*	β	*p*
Lysis	1,147	9.51	0.66	**0.002**	5,17	10.88	**<0.001**	1,16	0.11		0.740
Agglutination	1,146	2.91		0.090	5,16	3.85	**0.018**	1,16	3.52		0.079
Haptoglobin	1,147	0.35		0.554	5,16	4.38	**0.011**	1,16	9.47	−0.03	**0.007**
Body mass	1,148	113.57	4.40	**<0.001**	5,17	9.89	**<0.001**				
H/L ratio^a^	1,150	4.67	−0.28	**0.032**	4,153	0.91	0.462	1,152	0.04		0.850
Heterophils^a^	1,150	5.84	−0.28	**0.017**	4,153	1.80	0.133	1,152	0.11		0.745
Lymphocytes^a^	1,150	1.42		0.235	4,153	0.28	0.888	1,152	0.01		0.932
Basophils^a^	1,150	0.07		0.794	4,153	6.19	**<0.001**	1,152	0.50		0.479
Monocytes^a^	1,150	0.26		0.609	4,153	4.83	**0.001**	1,152	0.01		0.936
Eosinophils^a^	1,150	0.01		0.934	4,153	2.50	**0.045**	1,152	0.20		0.652

Individual bird identity was included as random effect to avoid pseudo replication whenever applicable. Results are from linear models after removing all non-significant terms (*p* > 0.05). *P* values <0.05 are bold

*BMd* body mass index that is calculated as each individual’s deviation from its corresponding season-, sex- and year-specific mean and thus independent of these three sources of variation

^a^Rows reporting data from generalized linear models

### Inconsistent seasonal patterns between years

The seasonal variation in immune parameters and body mass was not consistent between years. When we compared 2007 and 2008, we found a significant interaction between year and annual-cycle stage for all three immune variables (lysis, agglutination, haptoglobin) and body mass (Table 2; Fig. 3a–d). The interactions were not simply the result of 1 year that was higher and characterised by proportionally greater changes between stages (Fig. 3a–d), because they remained significant after log-transforming the response variables. The interaction sex × year was highly significant for lysis titres but not significant for agglutination, haptoglobin concentrations and body mass (Table 2). Males showed higher lysis than females in the first annual cycle, but lower than females in the second annual cycle. After removing the non-significant interactions from the model, there was no significant effect of sex on agglutination and haptoglobin (*df* = 1,290, *F* = 3.06, *P* = 0.08 and *df* = 1,292, *F* = 0.00, *P* = 0.97, respectively).

**Table 2:** Statistics and coefficients of the linear mixed models of measures of immune function and body mass in skylarks over two repeated annual cycles (2007 and 2008)

**Repeated annual cycle (2007, 2008)**										
	Sex × year			Annual-cycle stage × year			BMd			
	*df*	*F*	*p*	*df*	*F*	*p*	*df*	*F*	β	*p*
Lysis	1,47	9.41	**0.004**	4,47	5.27	**0.001**	1,46	0.74		0.393
Agglutination	1,46	0.34	0.564	4,48		**0.015**	1,47	0.89		0.350
Haptoglobin	1,42	0.73	0.399	4,47		**0.033**	1,47	7.22	−0.01	**0.010**
Body mass	1,43	0.04	0.843	4,44		**0.001**				

Individual bird identity was included as random effect to avoid pseudo replication. Results are from linear models after removing all non-significant terms (*p* > 0.05). *P* values <0.05 are bold. For explanation of *BMd* see footnote to Table 1

### Consistent patterns among annual cycles and individuals

The annual cycle as measured within single individuals (repeated measures) has the same basic pattern as at the population level. Seasonal patterns between territory establishment, breeding and moult measured on different individuals did not statistically differ from seasonal patterns established from repeated measurements within individuals for all immune parameters and body mass (always *P* > 0.22).

Regardless of which dataset was used (i.e. the complete 6-stage annual cycle of 2007, the repeated 5-stage annual cycles of 2007 and 2008, or the entire dataset including 2006–2009), post hoc analyses on models including season and year (if applicable) as main effects revealed that immune function differed consistently between breeding and autumn migration with respect to all five immune parameters that varied by stage (Tukey test always *P* < 0.029). Lysis and agglutination titres and haptoglobin concentrations were higher during breeding than during autumn migration; proportions of basophils and monocytes exhibited the opposite pattern. Also consistent among datasets, lysis titres were significantly higher during breeding than during spring migration (Tukey test always *P* < 0.001), and haptoglobin concentrations were significantly higher during breeding than during moult (Tukey test always *P* < 0.05).

### Variation among breeding seasons

Lysis, agglutination, haptoglobin and body mass showed significant differences among the four successive breeding seasons (Table 3). Lysis was significantly higher in 2007 than in all other years (Tukey test always *P* < 0.003; Fig. 2a). Agglutination was lower in 2009 than in 2007 and 2008 (Tukey test both *P* < 0.001), but did not differ from 2006 (Tukey test *P* = 0.66; Fig. 2b). Haptoglobin concentrations in 2006 were significantly higher than in all subsequent years (Tukey test always *P* ≤ 0.003) and concentrations in 2007 were significantly higher than in 2009 (Tukey test *P* = 0.008; Fig. 2c). Body mass in 2008 was lower than in 2006 and 2009 (Tukey test both *P* < 0.006; Fig. 2d). For the three immune variables and body mass, the differences among breeding seasons at the population level were qualitatively similar to the differences within individuals that were sampled in more than 1 year (always *P* > 0.18).

**Table 3:** Statistics and coefficients of linear mixed models of three plasma measures of immune function in skylarks during four consecutive breeding seasons (2006-2009) and of generalized linear models for leukocyte profiles which were measured only in one breeding season (2008)

**Breeding (2006–2009)**														
	Sex			Julian day				Year			BMd			
	*df*	*F*	*p*	*df*	*F*	β	*p*	*df*	*F*	*p*	*df*	*F*	β	*F*
Lysis	1,178	0.32	0.570	1,101	41.24	0.02	**<0.001**	3,101	24.98	**<0.001**	1,90	1.75		0.190
Agglutination	1,177	0.23	0.630	1,101	11.74	0.02	**0.001**	3,101	10.38	**<0.001**	1,95	0.24		0.628
Haptoglobin	1,181	0.74	0.391	1,96	0.88		0.350	3,97	13.19	**<0.001**	1,97	3.79	−0.01	0.054
Body mass	1,183	135.2	**<0.001**	1,98	30.01	−0.02	**<0.001**	3,98	5.86	**0.001**				
H/L ratio^a^	1,65	1.75	0.190	1,65	2.17		0.146				1,65	0.83		0.373
Heterophils^a^	1,65	1.55	0.217	1,65	1.85		0.178				1,65	0.73		0.395
Lymphocytes^a^	1,65	1.97	0.166	1,65	2.26		0.138				1,65	0.9		0.347
Basophils^a^	1,65	0.06	0.813	1,65	3.79		0.056				1,65	0.34		0.563
Monocytes^a^	1,65	0.02	0.889	1,65	4.05	0.02	**0.048**				1,65	0.32		0.573
Eosinophils^a^	1,65	1.64	0.206	1,65	0.06		0.801				1,65	1.25		0.267

Individual bird identity was included as random effect to avoid pseudo replication whenever applicable. Results are from linear models after removing all non-significant terms (*p* > 0.05). *P* values <0.05 are bold. For explanation of *BMd* see footnote to Table 1

^a^Rows reporting data from generalized linear models

### Variation within the breeding season

**Fig. 4:**
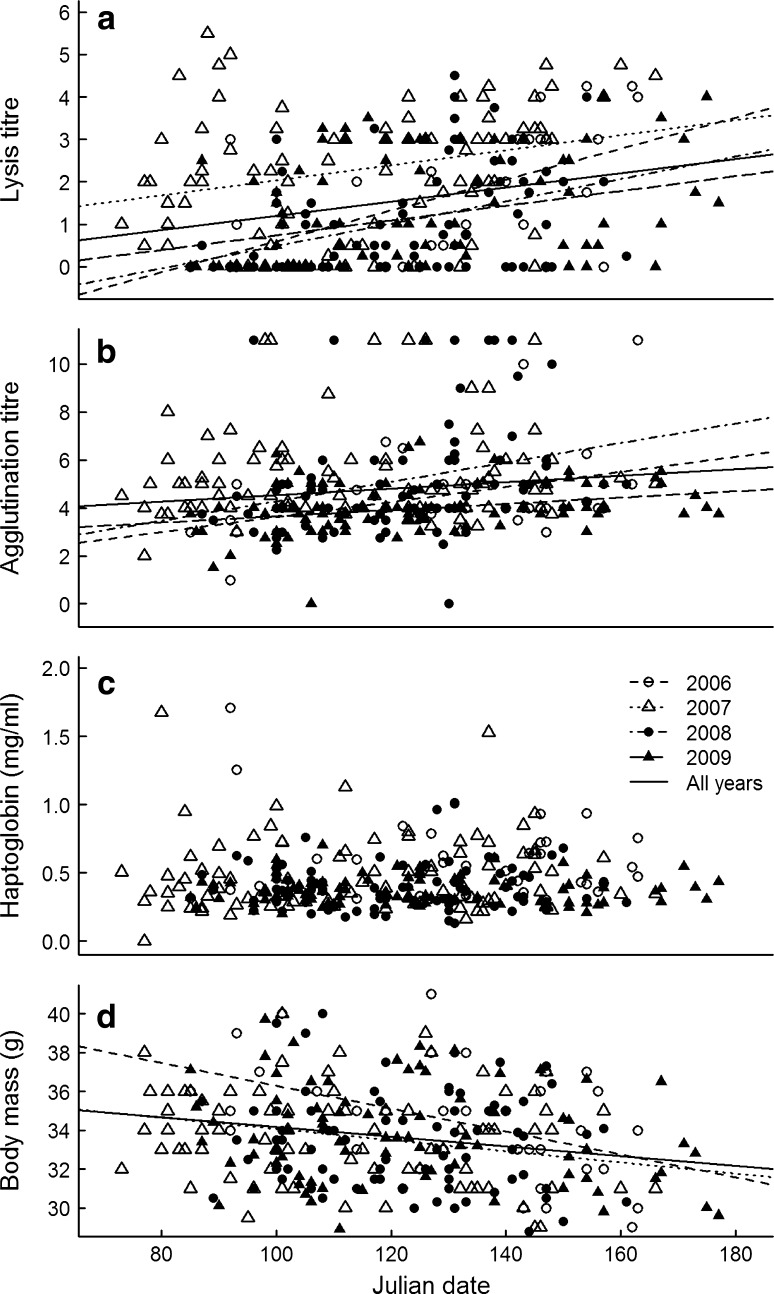
Patterns of** a** lysis titre,** b** agglutination titre,** c** haptoglobin concentrations and** d** body mass in the course of four different breeding seasons in free-living skylarks. Regression slopes are presented if* p* < 0.05

Over the course of four pooled breeding seasons, lysis and agglutination titres both increased, body mass decreased, and haptoglobin concentration showed no trend (Fig. 4a–d). The effect of Julian day was significant for lysis, agglutination and body mass (Table 3). This effect differed marginally between years for body mass (Julian day × year interaction *df* = 3,94, *F* = 2.78, *P* = 0.045), but did not differ between years for lysis and agglutination (Julian day × year interaction *df* = 3,87, *F* = 2.00, *P* = 0.12 and *df* = 3,92, *F* = 2.18, *P* = 0.10, respectively). In all four cases, the within-season pattern did not differ between birds sampled repeatedly and birds sampled only once (always *P* > 0.39). As the 2008 breeding season progressed, proportions of monocytes and basophils increased. The increase in monocytes was marginally significant, while the increase in basophils was marginally non-significant (Table 3). There was no relationship between Julian date and the H/L ratio or the proportions of heterophils, lymphocytes or eosinophils. During breeding, males and females did not differ significantly in any of the plasma indices or WBC counts (Table 3). All effects of date did not deviate from linearity, since date squared was never significant.

### Correlations between immune indices and body mass index

**Fig. 5:**
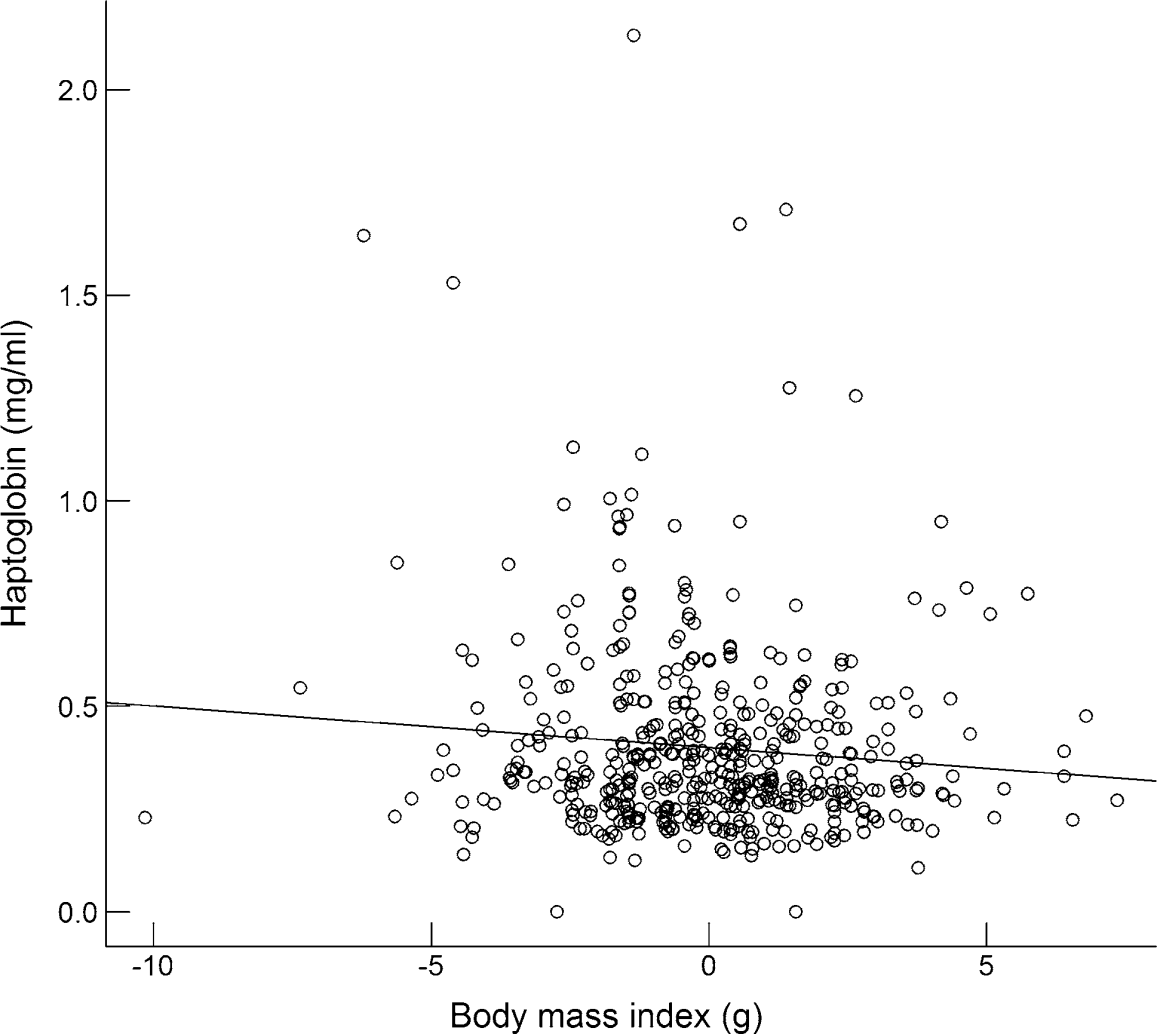
Relationship between a body mass index (in g) and haptoglobin concentrations in free-living skylarks. The body mass index is calculated as each individual’s deviation from its corresponding season-, sex- and year-specific mean and thus independent of these three sources of variation. Data from all years and annual-cycle stages pooled. Statistics for the separate datasets can be found in Table 1a–c

Individuals with a relatively high body mass had relatively low haptoglobin concentrations regardless of which dataset was used (Fig. 5). This relationship was significant for both the single and the repeated annual cycle (Tables 1, 2) and borderline non-significant (*p* = 0.054) during breeding (Table 3). We found no relationship with body mass index for lysis, agglutination or any of the WBC measures (Tables 1, 2, 3).

## Discussion

We found variation in indices of innate and acquired immune defence among six annual-cycle stages in free-living skylarks, but the shape of the seasonal patterns varied with immune index and differed between years. Body mass also showed variation among annual-cycle stages, and, as with the immune indices, the pattern differed between years. The among-year differences in body mass suggest that environmental factors such as food availability, temperature or pathogen pressure varied. Such factors could also lead to the differences in seasonal immune patterns among years. If prevailing environmental conditions and immune patterns are linked, then seasonal and annual variation within individuals (repeated measures) should parallel seasonal and annual variation measured at the population level; our results supported this. This finding also made it unlikely that aging explained any differences between years. Furthermore we found little evidence for differences between the sexes, and only haptoglobin concentrations were (inversely) related to body mass. Overall, our study supports the premise that birds seasonally modulate their immune system during the annual cycle. Because years differ, we conclude that this modulation is not a result of trade-offs dictated only by energetically-demanding, regularly-occurring annual cycle activities, but that environmental variation among annual-cycle stages and years is important.

### Seasonal patterns of immune function are flexible

Our finding of inter-annual differences in seasonal patterns of immune indices raises the question whether other species are similarly flexible between years and whether it depends on a species’ ecology. Other studies have suggested trade-offs between certain annual-cycle activities and immune function: reduced immune function has been described during migration (Owen and Moore 2008), moult (Moreno-Rueda 2010), winter (Svensson et al. 1998) and breeding (e.g. Ilmonen et al. 2000; Bonneaud et al. 2003; Tieleman et al. 2008), and Pap et al. (2010a, b) report seasonal variation over a complete annual cycle. These studies all considered only a single year, and the observed seasonal differences might also result from particular ecological conditions rather than physiological trade-offs that differed consistently between annual-cycle stages. Because we found differences in seasonal variation between years, we hypothesise that immune function is influenced by the combined effects of resource availability and parasite pressure, which in turn affect trade-offs between immune function and other fitness-related physiological systems. This process could lead to immunological flexibility among annual-cycle stages and among years. The fact that other fitness-related parameters like clutch size, breeding success and recruitment rate, which are known to be largely influenced by food availability and weather conditions, also vary considerably between years (Hegemann et al., unpublished data), supports our argument that environmental conditions differ between years. At the same time, it highlights that studies of trade-offs between immune function and other fitness-related processes need to incorporate variation in environmental conditions within and among annual-cycle stages and years.

During territory establishment, breeding and moult, we exclusively sampled birds from our local study population, but during winter, spring migration and autumn migration, we caught a mixture of local birds and birds from more northern and eastern locations. One might therefore ask if the variation in seasonality of immune function between years stems from sampling different proportions of birds from different origins during winter and migration in the 2 years. We cannot completely rule out this possibility, but it is unlikely that it explains the between-year variation, for two reasons: (1) within the breeding season and during moult we also observed variation between years, confirmed by repeated measurements on the same individuals; and (2) the three immune parameters and body mass were never consistently different within one stage between the 2 years. Only a subset of measured parameters differed, similar to patterns expressed by our local study population during breeding and moult.

Despite the flexibility of immune patterns, we also found consistent differences in immune function between some annual-cycle stages, indicating that some regularity in seasonal patterns of immune function exists. All five immune parameters that showed significant seasonal variation showed differences between breeding and autumn migration independent of which dataset we considered: lysis, agglutination and haptoglobin concentrations were higher during breeding, while monocyte and basophil proportions were higher during autumn migration. These consistent differences between breeding and autumn migration could indicate that some immune patterns are more dependent on seasonal changes in the species’ ecology. Going from breeding to autumn migration, skylarks switch their diet, become less aggressive and form flocks. The changes in WBC proportions might reflect this and other processes. For example, flocking may enhance the transmission of infectious diseases and/or parasites which could explain higher concentrations of basophils that are involved in parasite elimination (Latimer and Bienzle 2000). Likewise, phagocytic monocytes can play a role in removal of damaged cells (Latimer and Bienzle 2000), which might accumulate while actively migrating.

In summary, we see a tight connection between the hypothesis of immune responses being traded off with other annual cycle demands (Owen-Ashley and Wingfield 2007; Buehler and Piersma 2008; Martin et al. 2008), and the hypothesis that seasonal modulations result from seasonal environmental fluctuations in, e.g., disease risk and parasite exposure, food availability or weather conditions (Nelson et al. 2002; Møller et al. 2003; Hasselquist 2007). To further resolve the processes underlying seasonality in immune parameters, longer term monitoring of immune function in free-living birds is required. Additionally, experimental studies that manipulate environmental conditions or trade-offs in several annual-cycle stages (see Buehler et al. 2008b for an example on captive birds) and/or across multiple years can help us better understand the intra-annual changes in immune function. Because pathogen and parasite exposure can also shape the immune system, and their influence can exert effects over a wide range of timescales (among days or years, through evolutionary time, etc.), measures of pathogen pressure should be included where possible (Horrocks et al. 2011).

### Interpretation of immune indices and trade-offs within the immune system

In the current study, we focused on measures that are relatively insensitive to pathogen challenge (e.g. hemolysis and hemagglutination, Matson et al. 2005), that are known to change over the shortest of timescales but that typically return to baseline levels after a response is resolved (e.g. haptoglobin, Van de Crommenacker et al. 2010; and leukocyte distributions, Latimer and Bienzle 2000), or that are repeatable and bear some capacity to predict future responses (e.g. habtoglobin, Matson et al. 2012). Most of these measures represent the first lines of defence against infection. Lymphocytes are most closely associated with acquired immunity (second line of defence; Roitt et al. 1998), and natural antibodies, the main driver of agglutination, are best viewed as a link between the innate and acquired immune sub-systems (Ochsenbein and Zinkernagel 2000). Overall, this approach of measuring a suite of different indices that appear to be relatively insensitive to cumulative effects of exposure has proven useful in our current application and in other recent studies (Boughton et al. 2011; Buehler et al. 2011; Demas et al. 2011; Palacios et al. 2012).

The seasonal modulation and possible reorganisation of immune indices in the current study contrast with the year-round consistency of the inflammatory responses (acute phase responses to an endotoxin injection) exhibited by the same skylark population (Hegemann et al. 2012a). This contrast hints at different levels of regulation of the immune system. It also suggests that, while the ability to always respond by a certain magnitude to an infection may be crucial to survival, the starting points (i.e. the levels of immunological maintenance) required to initiate these responses differ among stages and indices. On the one hand, variable starting points may reflect a time-specific balance between the availability of resources and the likelihood of encountering a challenge. On the other hand, such responses may reflect the evolutionary pressure to address dire situations (e.g. a breach of initial defences by an infectious agent) with acute redirection of resources in an attempt to prevent mortality. In terms of methodology, this contrast highlights the utility of quantifying both maintenance and response components when studying wild animals.

### Variation within breeding seasons

As the breeding season progressed, lysis, agglutination and the proportion of monocytes and basophils increased and body mass decreased, but there was no trend in haptoglobin or the other white blood cell types. Increases in lysis and agglutination were measured in all 4 years and the slope of the increase did not differ between years even though the mean values did. These patterns were also established from individuals measured repeatedly; a result that diminishes the possibility that these trends result from different timing by individuals with different immune characteristics. Further studies are required to determine if the increases in lysis and agglutination with the progression of the breeding seasons result from increases in pathogen and parasite pressure, from a shift in the balance between current and future reproduction or are linked to other changes.

### Immune variation with sex and body condition

Some studies present hypotheses (Hasselquist 2007; Martin et al. 2008) or data (Parejo and Silva 2009; Pap et al. 2010b) suggesting that males and females differently allocate resources to the immune system due to their different behaviours and physiology. Other studies have found little or no evidence for sex differences in immune parameters (Raberg et al. 2003; Lee et al. 2006; Martin et al. 2006). Our results are in line with the second group: we found few sex differences in general, and we did not find any significant interaction between sex and annual-cycle stage, despite differences in physiology (e.g. reproductive organs, hormone profiles) and behaviour (male song flights and territorial defense, female incubation) between sexes in skylarks, especially during territory establishment and breeding.

Birds with relatively high haptoglobin concentrations had relatively low values of a body mass index, a measure of condition independent of season, sex and year. The slope of this relationship appears shallow, but changes of 0.1 mg/ml are biologically relevant (Van de Crommenacker et al. 2010). Acute increases in haptoglobin concentrations normally signify an infection-associated inflammation (Thomas 2000). Thus, underweight individuals with high haptoglobin concentrations might be suffering from systemic inflammation and its indirect behavioural or physiological effects that limit food intake or its direct effects of elevated metabolic rate (Owen-Ashley et al. 2006; Owen-Ashley and Wingfield 2007; Hegemann et al. 2012a). At the population level, haptoglobin concentrations were highest during territory establishment and breeding. During these stages, skylarks are territorial and aggressive (Glutz von Blotzheim and Bauer 1985; Donald 2004). The high risk of injury during territorial fights (little skin scratches when birds attack each other with their feet or bill) may be one of the causes for high haptoglobin concentrations, even though heterophil concentrations, another important line of first defence, were not specifically higher during these stages.

### Conclusion and future perspectives

To conclude, we found that indices of innate immunity and leukocyte distributions differ among the six distinct annual-cycle stages experienced by a free-living temperate zone bird. These differences in immune function and differences in body mass among annual-cycle stages were inconsistent between two study years. However, all within- and among-season patterns were similar at the levels of individual and population. Overall, our results suggest that immune function is sensitive to prevailing environmental conditions, which can differ not only from stage to stage but also from year to year. Our study highlights the need for measurements across multiple years in order to draw sound conclusions about seasonal and annual variation in physiology. Going forward, studies of this sort should strive to identify influential environmental factors (e.g. resource availability and pathogen pressure) and incorporate relevant measurements of these factors. Hence, we stress the importance of a detailed ecological knowledge of the studied species. Lastly, experimental studies that manipulate immune function will be useful for revealing and understanding any trade-offs that occur during an annual cycle.
